# THC and CBD affect metabolic syndrome parameters including microbiome in mice fed high fat-cholesterol diet

**DOI:** 10.1186/s42238-022-00137-w

**Published:** 2022-05-30

**Authors:** Jonathan Gorelick, Tal Assa-Glazer, Gil Zandani, Anna Altberg, Noa Sela, Abraham Nyska, Zecharia Madar

**Affiliations:** 1 Eastern Regional R&D Center, Kiryat Arba, Israel; 2 The Robert H. Smith Faculty of Agriculture, Food and Environment, The Hebrew University of Jerusalem, Rehovot, Israel; 3 Department of Plant Pathology and Weed Research, Volcani Center, Rishon LeZion, Israel; 4 Sackler School of Medicine, Tel Aviv University, Tel Aviv, Israel

**Keywords:** NAFLD, THC, CBD, Microbiome, Cannabis, Phytocannabinoids

## Abstract

**Background:**

Nonalcoholic fatty liver disease (NAFLD) is associated with metabolic syndrome, which often includes obesity, diabetes, and dyslipidemia. Several studies in mice and humans have implicated the involvement of the gut microbiome in NAFLD. While cannabis and its phytocannabinoids may potentially be beneficial for treating metabolic disorders such as NAFLD, their effects on liver diseases and gut microbiota profile have yet to be addressed. In this study, we evaluated the therapeutic effects of the two major cannabinoids, delta-9-tetrahydrocannabinol (THC) and cannabidiol (CBD), on NAFLD progression.

**Methods:**

NAFLD was induced by feeding mice a high fat-cholesterol diet (HFCD) for 6 weeks. During this period, the individual cannabinoids, THC or CBD, were added to the experimental diets at a concentration of 2.5 or 2.39 mg/kg. Profile of lipids, liver enzymes, glucose tolerance, and gene expression related to carbohydrate lipids metabolism and liver inflammation was analyzed. The effect of THC or CBD on microbiota composition in the gut was evaluated.

**Results:**

While not alleviating hepatic steatosis, THC or CBD treatment influenced a number of parameters in the HFCD mouse model. CBD increased food intake, improved glucose tolerance, reduced some of the inflammatory response including TNFa and iNOS, and partially mitigated the microbiome dysbiosis observed in the HFCD fed mice. THC produced a much weaker response, only slightly reducing inflammatory-related gene expression and microbiome dysbiosis.

**Conclusions:**

The results of this study indicate the potential therapeutic effects of individual phytocannabinoids are different from the effects of the cannabis plant possessing a mixture of compounds. While CBD may help ameliorate symptoms of NAFLD, THC alone may not be as effective. This disparity can putatively be explained based on changes in the gut microbiota.

## Introduction

Nonalcoholic fatty liver disease (NAFLD) is a global health issue with prevention/treatment a major challenge for world health services (Cotter and Rinella 2020). While the pathogenesis of NAFLD is not well understood, it involves the development of hepatic steatosis due to a complex interaction of metabolic dysfunctions usually including insulin resistance and dyslipidemia, which cause liver inflammation and injury, eventually leading to fibrosis, cirrhosis, and even carcinoma (Bertot and Adams 2016). Therapeutic approaches focus on lifestyle modification, with diet and exercise interventions the first line of therapy (Neuschwander-Tetri 2020). Considering the importance of diet on the development and treatment, it is not surprising that an increasingly important factor in the development of NAFLD is the gut microbiota (Kolodziejczyk et al. 2019), the collection of microorganisms including bacteria, fungi, archaea, etc. living in the gut. Relegated to obscurity until recently, the microbiota is quickly emerging as an important player in a wide array of physiological functions (Valdes et al. 2018). A number of animal (Le Roy et al. 2013; Zeng et al. n.d.) and clinical (Zhu et al. 2013; Zeng et al. 2021) studies support the microbiota’s involvement in the development of NAFLD.

While a variety of pharmacological approaches are currently being evaluated for the treatment of NAFLD, an important target that warrants more attention is the endocannabinoid system (ECS), a complex physiological system with two primary G-protein-coupled membrane receptors, CB1 and CB2, that are involved in a wide range of metabolic processes (Gatta-Cherifi and Cota 2015). *Cannabis* sp., from which the ECS derives its name, and the cannabinoids it contains have been shown to affect a wide variety of physiological functions (Ligresti et al. 2016) and have been identified as potential treatments for a number of diseases (Montero-Oleas et al. 2020) including metabolic syndrome (Assa-Glazer et al. 2020).

While cannabis possesses a vast number of bioactive compounds, the most well known and studied by far are THC followed by CBD. These two compounds have been the focus of a number of studies on a wide range of disease models including metabolic syndrome (Di Marzo et al. 2015; Jadoon et al. 2016b), although little work has been performed on their effects on NAFLD (Silvestri et al. 2015; Dibba et al. 2018).

### THC or CBD in vivo NAFLD mouse model

The high fat-cholesterol diet (HFCD) has proven its utility as a model of fatty liver disease in mice (Savard et al. 2013). HFCD induces steatosis and inflammation, increased levels of ALT, total cholesterol, and triglycerides within 6 weeks. In this work, the primary cannabinoids found in cannabis, THC, and CBD were evaluated for their effects on the HFCD mouse model of NAFLD.

## Materials and methods

The cannabinoids studied, Δ^9^-tetrahydrocannabinol (THC) and cannabidiol (CBD), were obtained from Sigma-Aldrich (Rehovot, Israel).

### Animals and diets

All experiments were approved by the Institutional Animal Care Ethics Committee of the Hebrew University of Jerusalem (AG-14922-2). Thirty-two male C57BL/6J mice, 5–6 weeks old, were purchased from the Harlan Laboratories (Jerusalem, Israel). Mice were randomly divided into four experimental groups: (1) mice fed normal diet (ND, *n* = 8); (2) mice fed high fat + 1%(w/w) cholesterol + 0.5%(w/w) cholate diet (HFCD, *n* = 8); (3) mice fed HFCD containing CBD (2.39 mg/kg) (HFCD + CBD, *n* = 8); and (4) mice fed HFCD containing THC (2.5 mg/kg) (HFCD + THC, *n* = 8). The diet compositions are presented in Table 1. Cannabinoids dissolved in ethanol were incorporated into the respective experimental diets. Comparable amounts of ethanol were used in the control diets. The mice were housed in a controlled environment (12/12 h light/dark cycle, 18–24 °C) with ad libitum access to food and water. At the conclusion of 7 weeks, animals were sacrificed, and tissues were harvested for further analysis.

**Table 1:** Experimental diets (g)

	Normal diet (ND)	High-fat cholesterol (HF-Cho)
Casein	140	248
L-Methionine	1.8	4
Corn starch	496	182
Maltodextrin 10	125	73
Sucrose	100	61
Cellulose	50	50
Soybean oil	40	48
Lard	0	260
Cholesterol	0	10
Cholic acid	0	0.5
Mineral mix S10022M	35	42
Vitamin mix V10037	10	12
Choline chloride	2.5	3
BHT	0.014	0.02
Total	1000.314	993.52

### Oral glucose tolerance test

Oral glucose tolerance test (OGTT) was performed after 6 weeks of the experimental period. Prior to the OGTT, the mice were fasted for 8 h and given D-glucose (3 g/kg body weight) by gavage. Blood drawn from the tail tip at 0, 30, 60, and 120 min after the glucose loading was used to monitor glucose levels by a glucometer (Optium Xceed).

### Tissue collection

The mice were fasted for 12 h, weighed, and then sacrificed by isoflurane inhalation. Blood was collected from the vena cava, and plasma was obtained by centrifugation at 5000 × g at 4 °C for 10 min and stored at –20 °C. Adipose and liver tissue were removed, weighed, frozen in liquid nitrogen, and stored at –80 °C. A small sample from the right lobe of each liver was placed in 4% formaldehyde for histological analysis. Ceca were removed and their contents collected for microbiota analysis. Analyses of serum lipid profiles for blood liver enzymes and metabolic profile were performed by the American Laboratories (Herzliya, Israel) using standard kits. Plasma insulin levels were measured by Rat/Mouse Insulin ELISA kit (Merck, Cat no. EZRMI-13K). Lipid quantification in liver tissue was carried out using Folch’s method (Folch et al. 1957).

### Histological examination and grading

Histological slides were prepared by Patholab (Rehovot, Israel). Formalin fixed tissues were embedded in paraffin, and serial sections (3–5 μm thick) were cut from each block and stained with hematoxylin and eosin (H&E). Histopathological changes were scored by Prof. A. Nyska (website: http://www.nyska.net), a board-certified toxicologic pathologist, using semiquantitative grading of five grades (0–4), taking into consideration the severity of the changes (Schafer et al. 2018): grade 0, no change seen; grade 1, minimal; grade 2, mild; grade 3, moderate; and grade 4, severe.

### Protein extraction and Western blotting

Total protein was extracted from the liver tissue with standard lysis buffer. Lysates were centrifuged at 20,000 × g for 15 min, and the protein concentration was determined by the Bradford method. Samples were subjected to 7.5–10% SDS-PAGE and transferred onto nitrocellulose membranes. Blots were incubated with primary antibodies: anti-rabbit AMPK, pAMPK (Thr-172), AKT, pAKT (Cell Signaling Technology), anti-CB1, anti-CB2 (Abcam), and then, after several washes, with secondary goat antibodies (Jackson Immuno Research Laboratories, West Grove, PA, USA). The immune reaction was detected by enhanced chemiluminescence, with bands being quantified by densitometry and expressed as arbitrary units. The band optical density was analyzed on an Image Lab system (Bio-Rad) and normalized to lane total protein content as visualized with Ponceau S solution (Sigma-Aldrich, USA) (Sander et al. 2019).

### Quantitative real-time PCR

Total RNA was isolated using TRI Reagent (Sigma-Aldrich, Rehovot, Israel), according to the manufacturer’s protocol. Complementary DNA was prepared with a High-Capacity cDNA Reverse Transcription Kit (Quanta BioSciences, Gaithersburg, MD, USA). Quantitative real-time PCR (RT-qPCR) was performed with the 7300 Real-Time PCR System (Applied Biosystems, Foster City, CA, USA), with specific primers. Quantitative changes in gene expression were determined by normalizing against 18S mRNA. Primers used are listed in Table 2.

**Table 2:** Primer sequences for qRT-PCR

Name	Forward	Reverse
18s	5′-ACCGCAGCTAGGAATAATGG-3′	5′-CCTCAGTTCCGAAAACCAAC-3′
CB1R	5′-CTACTGGTGCTGTGTGTCATC-3′	5′-GCTGTCTTTACGGTGGAATAC-3′
CD36	5′-TCCTCTGACATTTGCAGGTCTATC-3′	5′-AAAGGCATTGGCTGGAAGAA-3′
iNOS	5′-AGCTCCCTCCTTCTCCTTCT-3′	5′-TCTCTGCTCTCAGCTCCAAG-3′
PEPCK	5′-CAAACCCTGCCATTGTTAAG-3′	5′-TGCAGGCACTTGATGAACTC-3′
PPARα	5′-CTTCCCAAAGCTCCTTCAAAAA 3′	5′-CTGCGCATGCTCCGTG-3′
TNFα	5′-ACGTGGAACTGGCAGAAGAG-3′	5′-CCACAAGCAGGAATGAGAAGA-3′

### Metagenomics — 16S ribosomal RNA gene

The effects of each diet on the bacterial population in the gut microbiome was examined by analyzing the prokaryotic 16S ribosomal RNA gene (16S rRNA) which is approximately 1500 bp long and contains nine variable regions interspersed among conserved regions. These variable regions were subjected to phylogenetic classification, in diverse microbial populations. A two-step PCR-based method for preparing samples for sequencing the variable V3 and V4 regions of the 16S rRNA gene was used. Bacterial DNA was extracted from fecal samples using the DNeasy PowerSoil Kit (Qiagen) according to the manufacturer’s instructions. Each sample was then quantified with a Qubit 2.0 Fluorometer (ThermoFisher Scientific, Waltham, MA, USA) and diluted to a final concentration of 5 ng/ μl in 10 mM Tris at pH 8.5. The 16S library preparation was carried out as described in Illumina’s 16S sample preparation guide with minor modifications. PrimeStar HS DNA Polymerase Premix (Takara-Clontech, Mountain View, CA, USA) was used instead of the PCR enzyme. Sequences with 97% similarity were assigned to the same operational taxonomic units (OTU). OTUs of representative sequences at a similarity of 97% and their relative abundances were used to calculate and analyze rarefaction curves.

### Statistical analysis

Values are presented as mean ± SEM. Data were analyzed by unpaired 2-tailed Student’s *t*-test or by analysis of variance (one-way ANOVA) followed by Tukey–Kramer HSD post hoc test. The significance level was *p* < 0.05 for all analyses. The JMP 14 Pro software suites (SAS Institute, Cary, NC, USA) were used for the analyses.

## Results

### Effect of cannabinoids in mice fed high fat-cholesterol diet

#### Body weight gain, food intake, liver, and adipose tissue weight

**Fig. 1:**
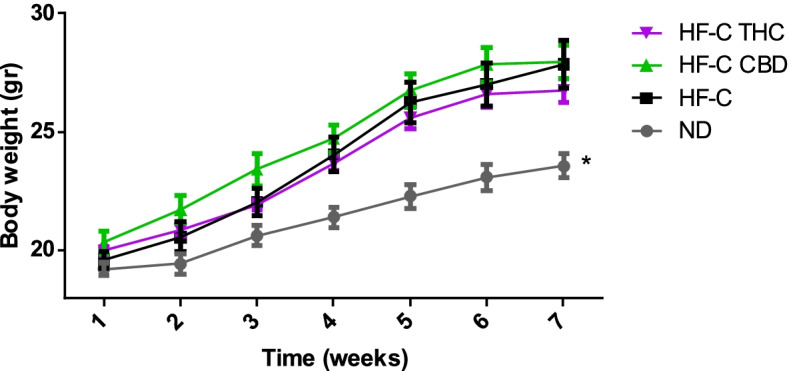
Effect of THC or CBD treatment on body weight gain in mice fed HFCD. Male C57BL/6J mice aged 7–8 weeks were fed either normal diet (ND, *n* = 8), high fat + 1%(w/w) cholesterol + 0.5%(w/w) cholate diet (HF-C, *n* = 8), HFCD containing CBD (2.39 mg/kg) (HF-C CBD, *n* = 8), HFCD containing THC (2.5 mg/kg) (HF-C THC, *n* = 8). Body weight was measured weekly over the experiment duration. All values are expressed as mean ± SEM. *(*p* < 0.05) according to Student’s *t*-test

**Fig. 2:**
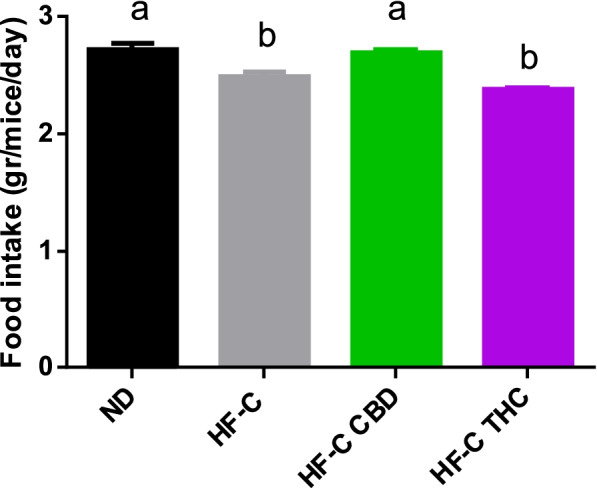
Effect of THC or CBD treatment on food intake in mice fed HFCD. Male C57BL/6J mice aged 7–8 weeks were fed either normal diet (ND, *n* = 8), high fat + 1%(w/w) cholesterol + 0.5%(w/w) cholate diet (HF-C, *n* = 8), HFCD containing CBD (2.39 mg/kg) (HF-C CBD, *n* = 8), HFCD containing THC (2.5 mg/kg) (HF-C THC, *n* = 8). Average food intake per mouse per day over the experiment duration is expressed as mean ± SEM. Columns marked with different letters (a, b) are significantly different (*p* < 0.05) according to the Tukey–Kramer post hoc test

**Fig. 3:**
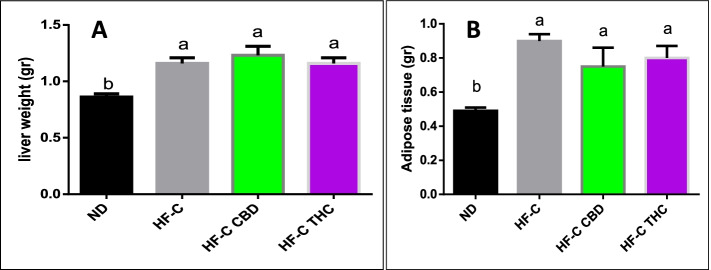
Effect of THC or CBD treatment on liver and adipose tissue weight in mice fed HFCD. Male C57BL/6J mice aged 7–8 weeks were fed either normal diet (ND, *n* = 8), high fat + 1%(w/w) cholesterol + 0.5%(w/w) cholate diet (HF-C, *n* = 8), HFCD containing CBD (2.39 mg/kg) (HF-C CBD, *n* = 8), HFCD containing THC (2.5 mg/kg) (HF-C THC, *n* = 8). Liver (**A**) or adipose (**B**) tissue weight of mice at the conclusion of the experiment is expressed as mean ± SEM. Columns marked with different letters (a, b) are significantly different (*p* < 0.05) according to the Tukey–Kramer post hoc test

All groups fed the HFCD displayed similar increases in body weight compared to the ND throughout the duration of the study (Fig. 1). Regarding daily food intake, mice fed HFCD consumed less food than the ND (Fig. 2). Interestingly, the HFCD-CBD group consumed more food than the other HFCD, almost comparable to the ND group. Neither cannabinoid affected liver nor adipose tissue size with an increase observed in all groups fed HFCD (Fig. 3).

### Effect on glucose tolerance

**Fig. 4:**
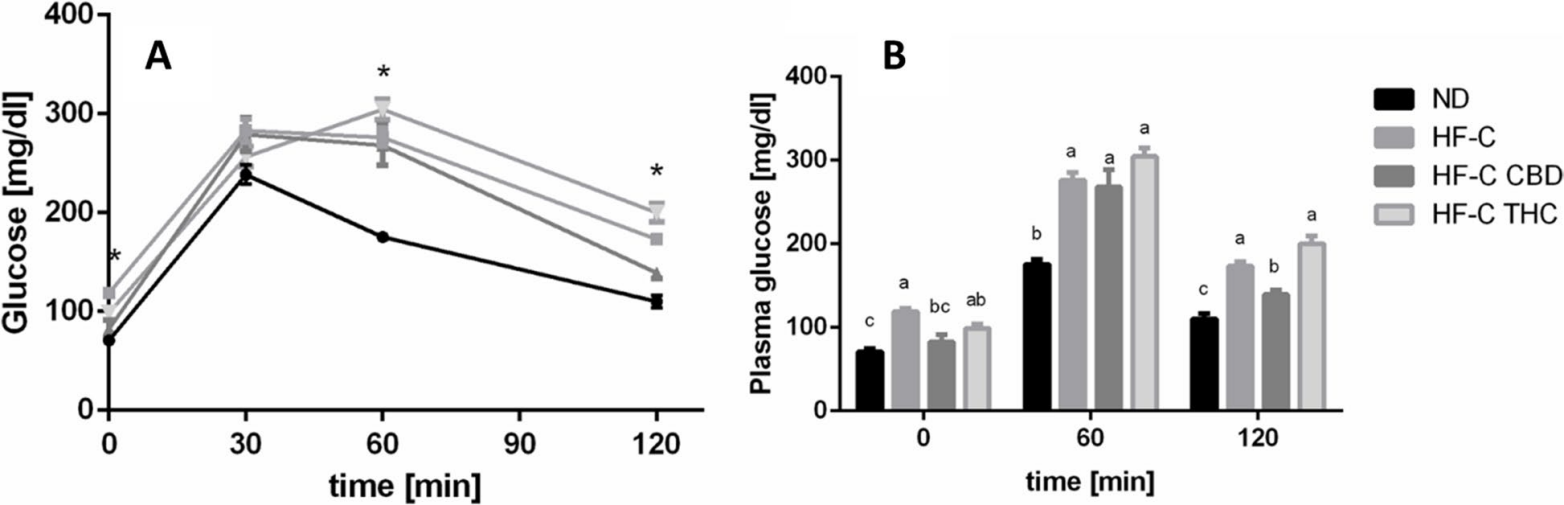
Effect of THC or CBD treatment on glucose tolerance levels in mice fed HFCD. Male C57BL/6J mice aged 7–8 weeks were fed either normal diet (ND, *n* = 8), high fat + 1%(w/w) cholesterol + 0.5%(w/w) cholate diet (HF-C, *n* = 8), HFCD containing CBD (2.39 mg/kg) (HF-C CBD, *n* = 8), HFCD containing THC (2.5 mg/kg) (HF-C THC, *n* = 8). After 5 weeks, oral glucose test tolerance was performed with values presented as mean ± SEM. **A** Results of glucose loading over time. *p < 0.05 using Tukey–Kramer test. **B** Hourly differences in glucose levels. Columns marked with different letters are significantly different (*p* < 0.05) using Tukey–Kramer test

While the HFCD increased fasting glucose levels, CBD supplementation mitigated the increase (Fig. 4). One-hundred twenty minutes after receiving oral glucose, blood glucose was significantly lower in CBD-treated mice compared to the HF-C.

### Effect on liver fat accumulation and histology

HFCD-induced hepatic steatosis and treatment with either THC or CBD produced no change in steatosis severity (Table 3) or in lipid fat accumulation in the liver (photos not shown).

**Table 3:** Effect of THC or CBD treatment on liver steatosis in mice fed HFCD

Group number	ND					HF-C					HF-C CBD						HF-C THC				
Treatment	Fed 6 weeks of normal diet					Fed 6 weeks of high-fat lard diet with 1% cholesterol and 0.5% cholic acid					Fed 6 weeks of high-fat lard diet with 1% cholesterol and 0.5% cholic acid and CBD						Fed 6 weeks of high-fat lard diet with 1% cholesterol and 0.5% cholic acid and THC				
Animal number	1	2	3	4	5	1	2	3	4	5	1	2	3	4	5	8	1	2	3	4	5
Organ	Liver					Liver					Liver						Liver				
**Hepatocytic vacuolation, consistent with fatty change — centrilobular**	0	0	0	0	0	3	3	3	3	3	3	3	3	3	3	3	3	3	3	3	3
**Inflammatory cell infiltration, multifocal**	0	0	0	1	1	2	2	2	2	2	2	2	2	2	2	2	2	2	2	2	2
**Hepatocellular necrosis, focal**	0	0	0	0	1	1	1	1	1	1	1	1	1	1	1	1	1	1	1	1	1
**Bile duct hyperplasia**	0	0	0	0	0	0	0	0	0	0	0	0	0	0	0	2	0	0	0	0	0

Male C57BL/6J mice aged 7–8 weeks were fed either normal diet (ND), high fat + 1%(w/w) cholesterol + 0.5%(w/w) cholate diet (HF-C), HFCD containing CBD (2.39 mg/kg) (HF-C CBD), HFCD containing THC (2.5 mg/kg) (HF-C THC). At the conclusion of the experiment, livers were collected for analysis. Steatosis grade was scored by the study pathologist, using semiquantitative grading of four grades (0–3), taking into consideration the severity of the changes (0, no lesion; 1, minimal change; 2, mild change; 3, moderate change)

### Effect on serum lipids profile and liver enzymes

Total cholesterol and HDL-cholesterol levels were high in all HFCD groups compared to ND, with neither of the cannabinoid treatments affecting these levels (Table 4). Triglycerides were lowered in all HFCD groups compared to ND with an increase observed in the CBD and THC-treated groups, although not statistically significant. Compared to the untreated HFCD animals, serum glutamic-oxaloacetic transaminase (SGOT) was lowered in the CBD-treated group.

**Table 4:** Effect of THC or CBD treatment on lipid and enzyme profile in mice fed HFCD

	ND	HF-C	HF-C CBD	HF-C THC
**Cholesterol (mg/dl)**	121.29 ± 5.73b	159.17 ± 3.94a	178.17 ± 9.31a	170.38 ± 3.9a
**HDL cholesterol (mg/dl)**	97.76 ± 3.76b	119.6 ± 4.2a	127.38 ± 9.05a	121.9 ± 3.09a
**Triglycerides (mg/dl)**	158 ± 14.05a	64.5 ± 5.4b	82 ± 3.59b	82.5 ± 2.89b
**Alk Phos (**μ**l/l)**	90.67 ± 2.59a	77.33 ± 6.44ab	73.67 ± 3.69b	77.75 ± 1.6ab
**SGOT (**μ**l/l)**	52 ± 5.47b	81.2 ± 4.91a	**67.5 ± 6.47ab**	72.25 ± 4.18a
**SGPT (**μ**l/l)**	30.67 ± 5.08b	61.6 ± 10.25a	54.33 ± 7.74a	57.25 ± 7.69a

All values are expressed as mean ± SEM (*n* = 5). Values marked with different letters are significantly different (*p* < 0.05) in the Tukey–Kramer test

*Alk Phos* alkaline phosphatase, *SGOT* serum glutamic-oxaloacetic transaminase, *SGPT* serum glutamic pyruvic transaminase

### Effect on carbohydrate metabolism genes in the liver

**Fig. 5:**
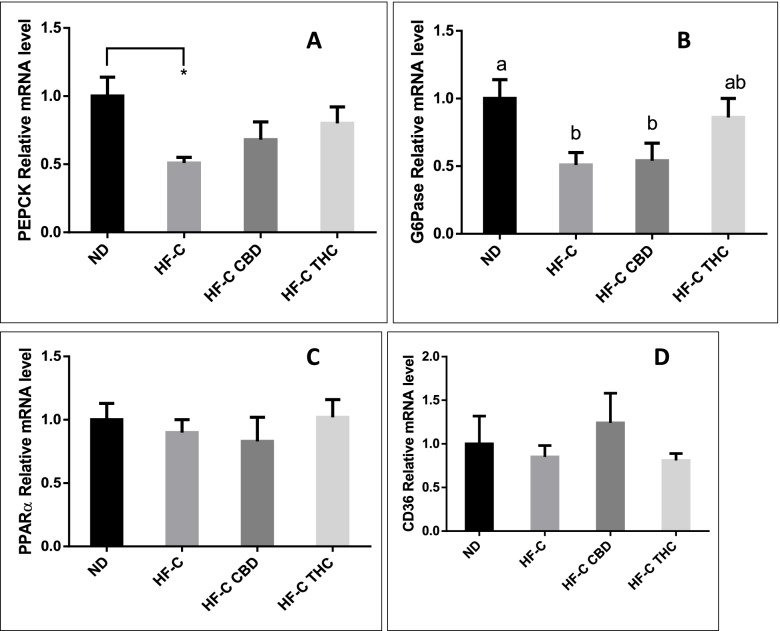
Effect of THC or CBD treatment on RNA expression of carbohydrate and lipid metabolism-related genes in mice fed HFCD. Male C57BL/6J mice aged 7–8 weeks were fed either normal diet (ND, *n* = 8), high fat + 1%(w/w) cholesterol + 0.5%(w/w) cholate diet (HF-C, *n* = 8), HFCD containing CBD (2.39 mg/kg) (HF-C CBD, *n* = 8), 4), HFCD containing THC (2.5 mg/kg) (HF-C THC, *n* = 8). At the conclusion of the experiment, RNA was isolated from harvested livers and gene levels quantified using qPCR. Values are expressed as mean of relative expression compared to ND ± SEM. Values marked with different letters are significantly different (*p* < 0.05) in the Tukey–Kramer test

**Fig. 6:**
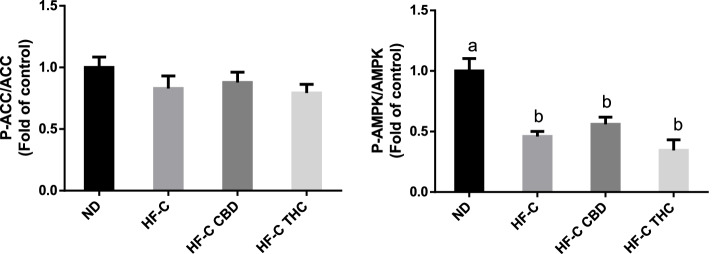
Effect of THC or CBD treatment on phosphorylation of carbohydrate and lipid metabolism-related proteins in mice fed HFCD. Male C57BL/6J mice aged 7–8 weeks were fed either normal diet (ND, *n* = 8), high fat + 1%(w/w) cholesterol + 0.5%(w/w) cholate diet (HF-C, *n* = 8), HFCD containing CBD (2.39 mg/kg) (HF-C CBD, *n* = 8), HFCD containing THC (2.5 mg/kg) (HF-C THC, *n* = 8). At the conclusion of the experiment, total protein was isolated from harvested livers and phosphorylated protein levels quantified. Values are expressed as phosphorylated/total protein ratio compared to ND ± SEM. Values marked with different letters are significantly different (*p* < 0.05) in the Tukey–Kramer test

Phosphoenolpyruvate carboxykinase (PEPCK) expression, which was significantly lowered in the HF-C group, was slightly increased in both the CBD and THC groups (Fig. 5A). G6Pase expression, which was similarly reduced by the HFCD, was increased in the THC group (Fig. 5B). No significant change was seen in the expression of CD36 (Fig. 5C) or PPARα (Fig. 5D) between the groups. At the protein level, the p-ACC/ACC ratio (Fig. 6A) did not change significantly in any of the groups, while the p-AMPK/AMPK ratio (Fig. 6B) was significantly reduced in all of the HFCD groups.

### Effect on inflammatory genes and endocannabinoid receptor expression in the liver

**Fig. 7:**
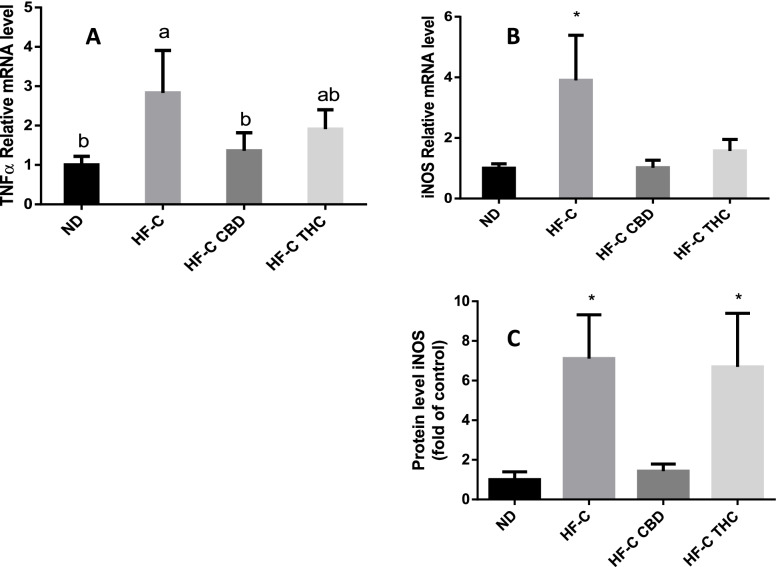
Effect of THC or CBD treatment on inflammatory gene expression in liver of mice fed HFCD. Male C57BL/6J mice aged 7–8 weeks were fed either normal diet (ND, *n* = 8), high fat + 1%(w/w) cholesterol + 0.5%(w/w) cholate diet (HF-C, *n* = 8), HFCD containing CBD (2.39 mg/kg) (HF-C CBD, *n* = 8), HFCD containing THC (2.5 mg/kg) (HF-C THC, *n* = 8). At the conclusion of the experiment, RNA was isolated from harvested livers and gene levels quantified using qPCR. Values are expressed as relative expression compared to ND (mean ± SEM). Values marked with different letters are significantly different (*p* < 0.05) in the Tukey–Kramer test. iNOS, inducible nitric oxide synthase; TNFa, tumor necrosis factor alpha

**Fig. 8:**
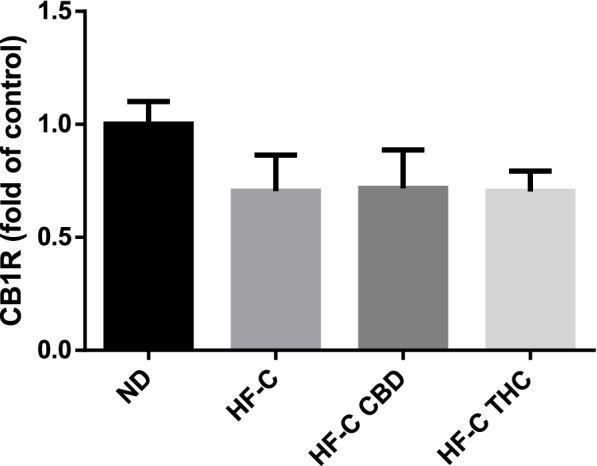
Effect of THC or CBD treatment on CB1 receptor gene expression in the liver of mice fed HFCD. Male C57BL/6J mice aged 7–8 weeks were fed either normal diet (ND, *n* = 8), high fat + 1%(w/w) cholesterol + 0.5%(w/w) cholate diet (HF-C, *n* = 8), HFCD containing CBD (2.39 mg/kg) (HF-C CBD, *n* = 8), HFCD containing THC (2.5 mg/kg) (HF-C THC, *n* = 8). At the conclusion of the experiment, RNA was isolated from harvested livers and gene levels quantified using qPCR. Values are expressed as relative expression compared to ND (mean ± SEM)

TNFα gene expression was increased by the HFCD, and both CBD and THC significantly mitigated this increase (Fig. 7A). Similarly, inducible nitric oxide synthase (iNOS) expression was elevated by the HFD, and both CBD and THC reversed this effect (Fig. 7B). Interestingly, only CBD treatment reduced the protein levels of iNOS (Fig. 7C). None of the treatments affected the expression of the CB1 receptor (Fig. 8).

### Effect on gut microbiota profile

#### Effects of HFCD and THC or CBD on microbiome

**Fig. 9:**
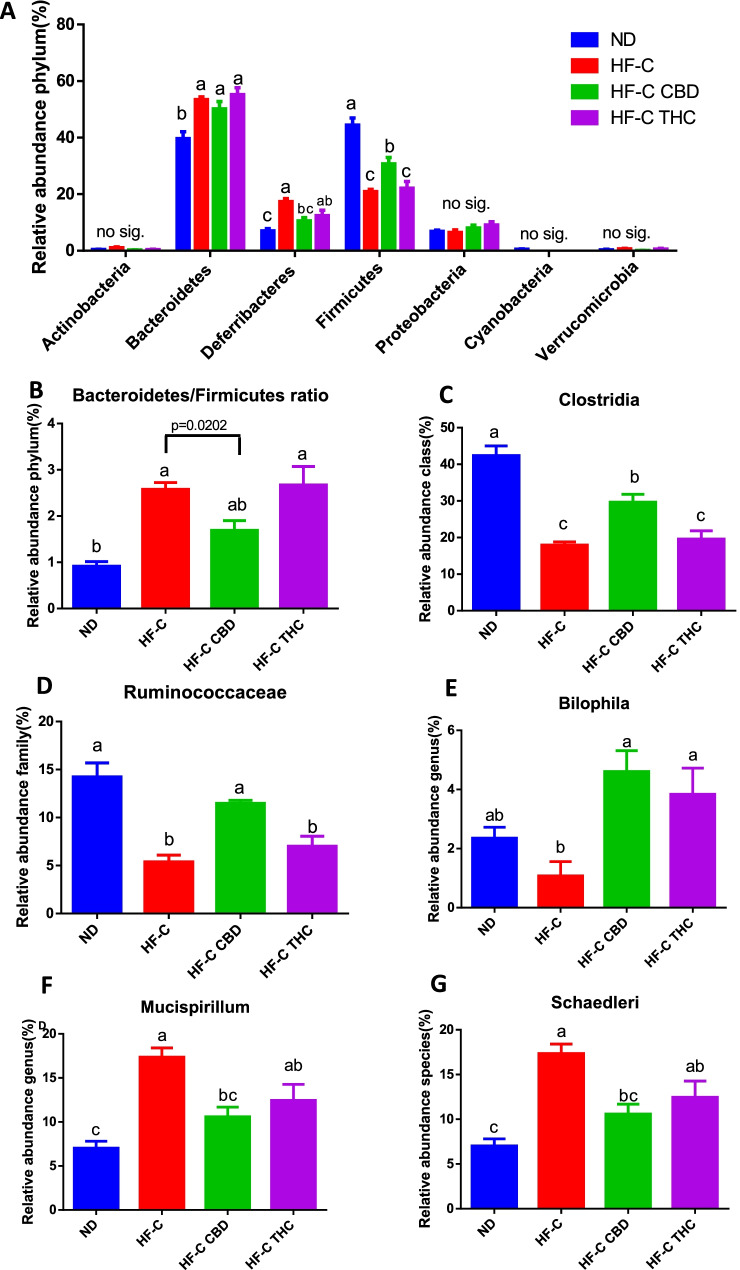
Effect of THC or CBD treatment on gut microbiota of mice fed HFCD. **A** Relative abundance of phyla. **B** Bacteroidetes to Firmicutes ratio. Relative abundance of **C** Clostridia, **D** Ruminococcaceae, **E***Bilophila*, **F***Mucispirillum*, **G***Schaedleri*. Male C57BL/6J mice aged 7–8 weeks were fed either normal diet (ND, *n* = 8), high fat + 1%(w/w) cholesterol + 0.5%(w/w) cholate diet (HFCD, *n* = 8), HFCD containing CBD (2.39 mg/kg) (HFCD+ CBD, *n* = 8), HFCD containing THC (2.5 mg/kg) (HFCD+THC, *n* = 8). Bacterial DNA was extracted, and values were expressed as mean ± SEM. Columns marked with different letters (a, b) are significantly different (*p* < 0.05) in the Tukey–Kramer test

The relative abundance of bacteria at the level of phylum, class, family, genus, and species was analyzed (Fig. 9). HFCD increased Bacteroides abundance with neither CBD nor THC producing any effect. HFCD also increased Deferribacteres abundance. However, both THC and CBD treatment reduced Deferribacteres levels, CBD even significantly (Fig. 9A). HFCD decreased Firmicutes abundance with CBD significantly increasing the levels, subsequently reducing the Bacteroidetes/Firmicutes ratio (Fig. 9B). No changes were observed in Actinobacteria, Proteobacteria, Cyanobacteria, or Verrucomicrobia. HFCD significantly reduces the abundance of Clostridia with the CBD treatment increasing the levels by 50% (Fig. 9C). This trend was observed regarding Ruminococcaceae (Fig. 9D) as well as with *Bilophila*, in which in addition to CBD, THC also increased the relative abundance (Fig. 9E). The inverse effect was observed on the genus level, regarding *Mucispirillum* (Fig. 9F) as well as the type species *Mucispirillum schaedleri* (Fig. 9G), in which HFCD increased the relative abundance and CBD attenuated this increase.

## Discussion

Overall, while not alleviating hepatic steatosis, THC or CBD treatment influenced a number of parameters in the HFCD mouse model of NAFLD including glucose tolerance and inflammation as well as the microbiome.

Surprisingly, CBD significantly increased daily food intake, restoring the levels to that of the ND. While CBD has been shown to inhibit the hyperphagia produced by cannabinoid receptor agonists (Scopinho et al. 2011), this is the first report of CBD increasing food intake. Also surprising was the lack of effect of THC, previously shown to increase food intake (Koch 2001). These discrepancies can at least partially be explained due to the chronic administration of high doses of cannabinoids compared to earlier work which involved primarily acute administration of significantly lower doses.

CBD’s influence on food intake did not produce any changes in total body, liver, or adipose tissue weight, suggesting that increased consumption was compensated for by changes in metabolic processes, reinforcing the important role of cannabinoids and energy homeostasis (Horn et al. 2018).

Regarding blood glucose, CBD significantly reduced both the fasting blood glucose levels and 120 min after receiving oral glucose (Fig. 4). This is in contrast with prior clinical studies (Jadoon et al. 2016b). Interestingly, in previous work, CBD was shown to slightly reduce blood glucose levels in healthy rats but not in rats fed a high-fat diet (Bielawiec et al. 2020). In contrast, THC treatment which lowered fasting glucose levels, produced a slight increase in blood glucose after OGTT, although not statistically significant. THC has been documented to both increase and decrease glucose uptake, seemingly dependent on location and dose (Miederer et al. 2017). These results further reinforce the important role of the ECS in modulating glucose uptake (Pagano et al. 2007).

Neither THC nor CBD reduced the severity of the liver steatosis or function in treated animals. Neither treatment altered the lipid or enzyme profiles produced by the HFCD with the exception of SGOT, which CBD at least partially restored (Table 3). Interestingly, CBD was shown to improve liver function in a mouse model of hepatic encephalopathy (Avraham et al. 2011), although the pathogenesis is quite different than in NAFLD.

While ineffective in significantly alleviating liver steatosis, treatment with CBD did reduce some of the markers of inflammation produced by the HFCD, including expression of TNFa and iNOS (Fig. 7), supporting CBD’s known anti-inflammatory activity. In a model of alcohol-induced liver steatosis, CBD significantly attenuated the alcohol feeding-induced increase in serum transaminase, hepatic inflammation (mRNA expressions of TNFα, MCP1, IL1β, MIP2 and E-selectin, and neutrophil accumulation), and oxidative/nitrative stress (lipid peroxidation, 3-nitrotyrosine formation, and expression of reactive oxygen species generating enzyme NOX2) (Wang et al. 2017a). Furthermore, CBD improved alcohol-induced hepatic metabolic dysregulation and steatosis by restoring changes in hepatic expression of ACC-1, FASN, PPARα, MCAD, ADIPOR-1, and mCPT-1. Other studies also support the potentially beneficial effects of CBD on the liver. CBD decreased cellular lipids in hepatocytes, in zebrafish larva, and in the liver of ob/ob mice (Silvestri et al. 2015). Interestingly, this effect was independent of CBR1 and TRPV1. While a comparable improvement in liver function was not observed in our study, this discrepancy can be at least partially explained due to differences in animal models (genetic vs. nutritionally induced) and route of administration (oral gavage vs. feeding).

### Microbiome

Supporting previous work, the HFCD significantly altered the microbiome composition at the phylum level, modulating the abundance of Bacteroidetes, Deferribacteres, and Firmicutes (Fig. 9A). The nature of this alteration and its relevance to disease development is a subject of discussion (Magne et al. 2020). The Bacteroidetes/Firmicutes ratio was higher in HFCD groups, consistent with previous studies showing elevated Bacteroidetes/Firmicutes ratio in NASH patients (Wong et al. 2013; Sobhonslidsuk et al. 2018). CBD treatment significantly reduced the ratio, although the importance of this ratio within the context of NAFLD is the source of contention with a number of conflicting reports which suggest that NAFLD may actually lower the Bacteroidetes/Firmicutes ratio (Porras et al. 2018; Mouzaki et al. 2013; Wang et al. 2016). This seeming contradiction may at least partially be due to other confounding factors within the context of metabolic disorders such as inflammation which can influence the microbiome (Silvestri et al. 2020).

Many of these inconsistencies can be resolved when looking at the changes in the microbiome at a higher resolution than just the phyla. HFCD lowered the abundance of Clostridia, Ruminococcaceae, and *Bilophila* (Fig. 9C–E). This was somewhat surprising as studies conducted on mice fed high-fat diet showed an increased abundance of Clostridia (Porras et al. 2017; Wang et al. 2017b). This discrepancy can at least be partially explained due to differences within the class. Higher levels of several Clostridia-related species are often associated with inflammation and pathogenic conditions (Maukonen et al. 2006; Rao et al. 2014; Severity 2017), while others may be protective against various allergens and related factors known to reduce intestinal permeability (Zou et al. 2018; Stefka et al. 2014; Seo et al. 2013). Interestingly, CBD increased Clostridia abundance, although it is unclear which species were effected and therefore difficult to understand the subsequent importance of this effect on NAFLD.

Ruminococcaceae, a family of gram-positive bacteria commonly found in the intestines of mammals, have the ability to break down cellulose and hemicellulose from plant material (Kim et al. 2019) and produce short-chain fatty acids (SCFAs). SCFAs are linked to a number of health benefits, and decreased SCFAs is a very important pathogenic factor in NAFLD (Wang et al. 2016). Our observations support previous studies in which patients with persistent NAFLD displayed significantly lower levels of Ruminococcaceae compared with healthy individuals (Zhu et al. 2013; Kim et al. 2019). CBD treatment significantly increased Ruminococcaceae abundance (Fig. 9D), possibly increasing the levels of SCFAs, and mitigating the development of NAFLD, although this remains to be verified.

The reduction of *Bilophila* abundance observed in the HFCD (Fig. 9E) seems to contradict previous studies where increased *Bilophila* was found in NAFLD patients (Jiang et al. 2015). Again, this discrepancy may be due to differences at the species level as well as location. *Bilophila wadsworthia* was shown to promote inflammation, intestinal barrier dysfunction, and bile acid dysmetabolism, leading to hepatic steatosis (Natividad et al. 2018). *Bilophila* species in adipose tissue may positively contribute to blood glucose control (Anhê et al. 2020) and possibly other factors related to NAFLD, although this needs further study. The implications of CBD and THC treatments resulting in higher levels of *Bilophila* remains to be determined.

The HFCD significantly increased the relative abundance of the genus *Mucispirillum* as well as the species, *Mucispirillum schaedleri*, with CBD treatment resulting in a significant reduction (Fig. 9F–G). *M. schaedleri*, a low-abundant member of the intestinal microbiota (Herp et al. 2019) which inhibits the intestinal mucus layer of rodents and other animals (Loy et al. 2017), is associated with inflammation in mouse models, and its increased abundance is correlated with the development of NAFLD (Zhang et al. 2020). The reduction in *M. schaedleri* caused by CBD may provide a hint of the mechanisms involved, although further work is needed.

While the exact role of THC or CBD in modulating the microbiome in the context of NAFLD is still not clear, a clear change was produced, which must be taken into consideration in any future treatment development. What remains to be determined is whether alterations of the microbiome are actually the cause of the other changes observed or only associated effects. Of course, this ambiguity can be resolved through fecal transplant studies which should be performed in the future. In addition, a greater understanding of the role of the endocannabinoid system regarding the gut microbiome and metabolic disorders is needed (Iannotti and Di Marzo 2021).

Interestingly, the beneficial effects of THC or CBD on the microbiome are not confined to metabolic-related pathologies. The ameliorating effects of a combination of THC and CBD on a murine model of experimental autoimmune encephalomyelitis (EAE) were linked to changes in the microbiome, specifically reduction in mucin degrading species such as *Akkermansia muciniphila* which were increased in the gut of EAE mice (Al-Ghezi et al. 2019). Fecal material transfer experiments confirmed that the THC + CBD-mediated changes in the microbiome play a critical role in attenuating EAE.

The potential use of cannabis or the isolated cannabinoids like THC or CBD for treatment of NAFLD remains a topic of debate. A recent observational study demonstrated an inverse association between cannabis use and NAFLD among adults in the USA (Adejumo et al. 2017). This observation was strengthened by a subsequent clinical trial in which the incidence of liver steatosis was significantly lower in cannabis users (Vázquez-Bourgon et al. 2019). On the other hand, a conflicting study showed no evidence that cannabis consumption reduces the risk of NAFLD (Wang et al. 2020). These discrepancies can possibly be explained by the changing chemical profiles found in different cannabis samples. The results from our study suggest that CBD may be more effective than THC at ameliorating NAFLD. Unfortunately, in the majority of studies involving cannabis, especially observational ones, the levels of THC and CBD are not defined. In addition to THC and CBD levels which can vary drastically between different varieties, there are a number of other bioactive compounds which can fluctuate due to various genetical and environmental factors (Bernstein et al. 2019).

In this work, CBD’s and to a lesser extent THC’s ability to ameliorate some of the parameters of NAFLD including microbiome dysbiosis were documented. While the preclinical work is promising, CBD’s efficacy in treating metabolic disorders remains to be proven (Jadoon et al. 2016a). Although cannabis and the cannabinoids found therein may produce a protective effect against NAFLD and liver steatosis, further work is needed to more clearly elucidate their potentially therapeutic effects.

## Data Availability

The datasets used and/or analyzed during the current study are available from the corresponding author on reasonable request.
